# The Impact of a Hypoallergenic Diet on the Control of Oral Lesions in Cats: A Case Report

**DOI:** 10.3390/ani14182656

**Published:** 2024-09-12

**Authors:** Luiza da Silva, Taís Martins, Mariana Yukari Hayasaki Porsani, Fabio Alves Teixeira

**Affiliations:** 1 Independent Researcher, Florianópolis 88052-629, Brazil; luh.silva@gmail.com (L.d.S.); taaismartins@gmail.com (T.M.); 2 NutricareVet, São Paulo 05690-080, Brazil; mariporsani@hotmail.com; 3 School of Veterinary Medicine and Animal Science, University of São Paulo-Brazil, São Paulo 05508-270, Brazil; 4 National Association of São Paulo for Small Animal Practice-Brazil, São Paulo 03077-000, Brazil

**Keywords:** oral health, stomatitis, gingivitis, food reaction, hypoallergenic diet

## Abstract

**Simple Summary:**

Feline gingivostomatitis is a common condition affecting the oral cavity of cats, characterized by severe mucosal inflammation. The etiopathogenesis remains not fully clear, but it is known to be multifactorial, involving alterations in immune response which may be related to infectious agents. Other potential triggers include food sensitivity and genetic and environmental factors. This study followed a cat refractory to conventional treatment who was placed on an elimination diet. After the implementation of the elimination diet, the cat showed remission of oral lesions. This report aims to highlight the importance of recognizing food sensitivity as a significant factor in maintaining the chronicity of the disease and how dietary management and switching to a hypoallergenic diet can aid in controlling the lesions. To the authors’ knowledge, this is the first documented case demonstrating the impact of food sensitivity on the progression of feline gingivostomatitis. The findings emphasize the importance of incorporating nutritional considerations into the comprehensive management of this challenging condition.

**Abstract:**

Feline stomatitis or gingivostomatitis is a chronic inflammatory condition affecting approximately 0.7% of patients. The cause is multifactorial, involving infectious agents, genetic factors, and environmental influences. Therapeutic strategies include pharmacological and surgical interventions to controlling inflammation and enhancing patient quality of life. There are discussions in the literature regarding the potential involvement of adverse food reactions as a contributing factor to oral cavity lesions, without clear evidence. This case report describes the control of gingivostomatitis with a hypoallergenic diet in a cat that had oral lesions and who was refractory to conventional treatment with prednisolone and cyclosporine, even after periodontal treatment and partial tooth extraction. After 30 days of dietary change, there was complete remission of the lesions. The animal was then re-exposed to the previous food, with new lesions appearing after 7 days. Upon returning to the hypoallergenic food, there was new remission of the lesions. This report suggests that food sensitivity may play a role in the control of feline gingivostomatitis, as periods of hypoallergenic diet coincided with the remission of the condition, even without changes in medication. It reinforces the importance of investigating adverse food reactions as clinical signs in the oral cavity of cats.

## 1. Introduction

Stomatitis is a complex, chronic, and destructive inflammatory process that affects the epithelium and lamina propria, often extending to submucosal tissues. Feline gingivostomatitis is a condition that affects the oral cavity of cats, characterized by severe inflammation in the mucosa. The lesions are ulcerative and proliferative and are frequently located in areas near the premolar and molar teeth, but they can extend to the palate, tongue, and pharynx, and in some cases, resorptive dental lesions are observed. It is estimated that this inflammation of the oral mucosa affects 0.7% of cats that receive veterinary care [1,2,3,4,5].

The etiopathogenesis is still not fully clear, but it is known to be multifactorial, involving alterations in immune response associated with the production of T lymphocytes and immunoglobulins, which may be related to infectious agents such as viruses (feline leukemia, feline immunodeficiency, calicivirus, herpesvirus, and panleukopenia) and commensal bacteria of the oral cavity [6,7,8]. One of the main causes of the disease’s chronicity is the exposure of the oral mucosa to agents present in dental plaque, increasing the severity of lesions [4,6]. Other possible triggers are related to food sensitivity or genetic and environmental factors [8].

Clinical signs vary according to the severity of lesions in the oral mucosa, but may include halitosis, ptyalism, hyporexia, anorexia, dysphagia, and behavioral changes due to pain, such as aggression and isolation. Subsequently, the patient presents progressive weight loss and dehydration, and the patient’s body initiates a process of proteolysis and lipolysis [3,4,9].

On oral physical examination, the inflammatory process in the mucosa can be observed. Complementary exams are necessary to rule out systemic conditions and exclude differentials that cause oral ulcerations [1,2]. Histopathologically, hyperplasia of the oral epithelium with deep ulcerations and an infiltrate of inflammatory cells is evident [10].

Inflammation control is most commonly achieved when clinical and surgical treatments are combined, aiming for an improvement in quality of life rather than a cure for the disease [3,10,11,12]. Surgical treatment consists of dental prophylaxis and extractions. In partial extraction, molar and premolar teeth are removed, while in total extraction, all teeth are removed. This intervention aims to minimize the contact of the oral mucosa with dental plaque and immune stimulation [12]. Clinical treatment aims to control pain, reduce inflammation and infection, and promote immunosuppression [3].

Regardless of the cause, nutritional management in these cases is fundamental since, with oral compromise, there is a high chance that the patient will not eat adequately. Moreover, food hypersensitivity is cited [1] as a potential trigger for gingivostomatitis. However, no evidence was found in the literature that dietary change aids in controlling oral mucosa inflammation. The objective of this study is to report a case of oral inflammation control in a feline using an extruded hypoallergenic diet based on hydrolyzed protein.

## 2. Case Presentation

**Figure 1:**
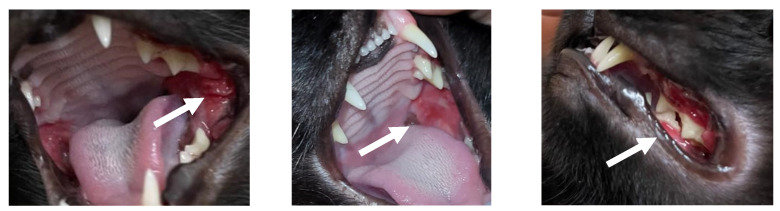
Lesions in the oral mucosa at the first appointment (arrows).

A domestic short hair feline patient, 2-year-old spayed female, living indoors, Feline Immunodeficiency Virus (FIV) and Feline Leukemia Virus (FeLV) negative, was presented with hematuria and dysuria. Despite these issues, the patient exhibited normal appetite, normal thirst, and was active. The diet consisted of a maintenance dry extruded diet with the main ingredients including chicken meat, poultry viscera meal, dehydrated egg, fish meal, isolated pork protein, potato starch, and cassava starch. On physical examination, a body condition score of 6/9 [13], lean mass score of 3/3 [14], healthy coat, and vital parameters within reference intervals were observed. However, upon examining the oral cavity, ulcerated lesions were noted in the caudal buccal mucosa, along with gingivitis on the upper premolar teeth (Figure 1).

Other conditions were ruled out since the parameters of the complete blood count, urea, creatinine, alanine aminotransferase, aspartate aminotransferase, total proteins, and albumin were all normal. Urinalysis showed struvite crystals, the urine culture and sensitivity tests were negative, and the abdominal ultrasound was suggestive of cystitis. After evaluating these results, and no signs to justify another cause of stress, we determined that the patient was likely experiencing stress-induced inflammatory cystitis due to discomfort from ulcers in the oral cavity. There are no other diagnostic indicators, such as histopathological evaluation, due to the owner’s financial issues.

**Figure 2:**
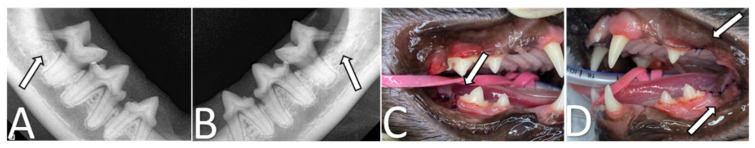
(**A**) Radiograph of the right mandible and (**B**) left mandible with bone loss in the region of the distal root of the first molar (arrows). Images (**C**) and (**D**) show exodontia. Arrows indicate the exodontia.

At the first appointment (day 1), prednisolone (1 mg/kg/SID, orally) and amitriptyline (2.5 mg/SID, orally) were prescribed. After 8 days of clinical treatment, the patient showed complete improvement of urinary symptoms, but the oral cavity lesions remained unchanged. Starting on the tenth day, the dose of prednisolone was gradually reduced by half (0.5 mg/kg SID) for 5 days and then administered on alternate days for an additional 5 days until the medication was completely discontinued. On the tenth day of treatment, cyclosporine (7 mg/kg/SID, orally) was prescribed, and after 14 days with cyclosporine, surgical treatment was performed for oral prophylaxis and extraction of teeth with gingivitis and bone resorption (Figure 2).

**Figure 3:**
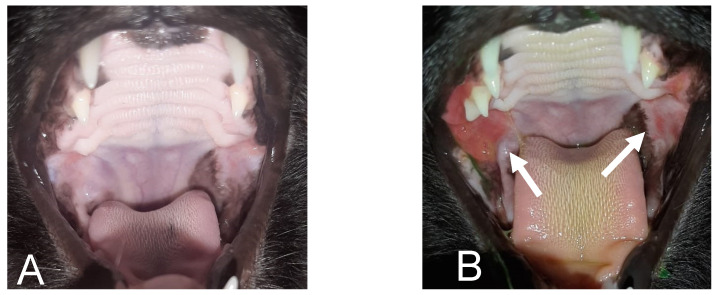
(**A**) Remission after 30 days with the use of the hypoallergenic diet. (**B**) Reappearance of lesions approximately 7 days after food re-exposure. Arrows indicate the location of the lesions.

Even after 30 days following the procedure, during which three molars were removed (Figure 2) and the patient was six weeks on cyclosporine, the cat continued to have the same oral lesions. We decided to replace the previous diet with a hypoallergenic extruded dry food based on hydrolyzed soy protein and rice bran (Hypoallergenic Feline, Royal Canin), prescribed according to maintenance energy needs [NEM (kcal/day) = 75 × BW^0.67^, where BW = body weight [15]]. After 30 days, the patient showed complete remission of the lesions (Figure 3A), and the amitriptyline was discontinued. At this point, the cat owner, who had another cat, requested to resume the maintenance diet, because they wanted both cats to eat the same diet. Therefore, the diet re-exposure was performed, without modifying the cyclosporine regimen, using the previously administered extruded dry food. After seven days, the patient exhibited inflammation of the oral mucosa again (Figure 3B), prompting a return to the hypoallergenic diet, after which the patient showed complete improvement of the lesions within a few days.

After remission of the oral lesions, the dosage of cyclosporine was reduced. This resulted in the recurrence of the lesions, making it necessary to return to the initial dose. After 6 months, the patient remained stable with the daily administration of cyclosporine and the hypoallergenic dry diet, without access to any other diet. Additionally, it was recommended to the caretaker to strictly control the amount of food to prevent the patient from gaining weight and becoming significantly overweight, which would worsen the feline’s condition.

## 3. Discussion

This case demonstrates that although conventional clinical and surgical treatments were combined, as described in the literature, the patient remained refractory. Since gingivostomatitis involves alterations in the immune response, a transition to a hypoallergenic diet was implemented, resulting in complete remission of the lesions.

Initially, the patient was medicated with prednisolone to reduce the inflammatory process, as it is effective in 70 to 80% of stomatitis cases [3]. Lower urinary tract signs could be justified by stress and pain, and it was managed with amitriptyline, a tricyclic antidepressant with anti-inflammatory, analgesic, sympatholytic, antihistaminic, and anticholinergic properties [16]. On the eighth day of treatment, despite the remission of urinary signs, the oral ulcerative lesions persisted. Specific periodontal treatment was performed, which is estimated to have an 80% success rate due to a reduction in antigenic stimulation [4]. Additionally, cyclosporine was administered, which is classified as an immunosuppressant that inhibits T lymphocyte activation by reducing pro-inflammatory cytokines [9]. After 44 days of medication use, improvement was expected, especially since the surgical treatment had already been performed [12]. Cyclosporine has an average time of 2 to 6 weeks before the onset of improvement in lesion cases. However, the patient did not show improvement in the lesions, even after surgical treatment.

Some authors [1,8,17] suggest that nutritional management similar to that approached in cases of adverse food reaction is relevant in these situations; however, there are no studies or case reports in the literature that clearly demonstrate this relationship between oral lesions and food hypersensitivity. The main clinical signs of food sensitivity in felines are cutaneous and gastrointestinal [18,19], with reports of ophthalmic, respiratory, and behavioral alterations [20,21,22].

The diagnosis and treatment of adverse food reactions are based on the use of an elimination diet followed by provocative exposure [23], as in the case reported here. For cutaneous clinical signs, the recommended elimination period is up to eight weeks [24]. For gastrointestinal clinical signs, this elimination diet time is not fully established, with some authors recommending a shorter time than for dermatological changes, such as 2 to 8 weeks [22,23,25]. The recommended provocative diet period for diagnostic confirmation of an adverse food reaction is one to two weeks in felines [26]. In the case reported here, the time taken for the elimination diet to result in an improvement in clinical signs and for the provocative test to result in recurrence of the lesions was in line with the time recommended in the literature. It is important to note that provocative exposure was only possible because the patient had an excellent appetite and no refusals during the diet changes, and because of the consent of the owner.

Since there was a dietary change and the patient was maintained exclusively on a hypoallergenic diet, leading to the first complete improvement of the ulcerative lesions in the oral mucosa without changes in the medication or environmental protocols, it is possible to consider that the patient had an adverse reaction to the food, manifested by lesions in the oral cavity. We raised the hypothesis that chronic antigenic stimulation caused by some component of the diet food was a cofactor in perpetuating the inflammatory process. The diet with hydrolyzed protein may have aided this process, as hydrolysis reduces the molecular weight of the protein, decreasing the likelihood of epitope recognition by the immune system, which could potentially trigger an adverse food reaction [27].

It is an unknown variable whether the cyclosporine took time to reach its maximum effect and whether this occurred during the elimination diet. However, the patient’s re-exposure to the previous maintenance diet and the subsequent recurrence of oral mucosal lesions, followed by new remission upon returning to the hypoallergenic food, increase the evidence that diet may be an important factor in controlling this patient’s inflammatory process.

In parallel with veterinary medicine, similar conditions can be observed in humans, where recurrent aphthous stomatitis (RAS) presents a multifactorial cause and a pathophysiology similar to feline gingivostomatitis. It is linked to genetic, environmental, and immunological factors, and can also be associated with food allergy [28,29]. Treatment of RAS in humans depends on the frequency, size, and number of the ulcers, and consists of topical and systemic treatment with corticosteroids and immunosuppressants [30,31]. A possible link between food allergy and some cases of RAS has been suggested, with about 25 to 75% of human patients experiencing remission of oral cavity lesions after following an elimination diet for 1 to 2 weeks [32].

In this reported case, we decided to maintain the feline patient on a combined regiment of the hypoallergenic diet and cyclosporine, as the initial attempt to discontinue the treatments individually was unsuccessful. Studies show that some allergic conditions in pets with cutaneous manifestations can be minimized with the use of hypoallergenic food based on hydrolyzed protein, but only when combined with medication during the initial stage of the diet trial [32]. However, it is uncertain how a new attempt to suspend the medication or change the diet would fare after a long period in our case.

In the “Guideline Proposal for the Management of Chronic Gingivostomatitis in Cats” [33], the authors note that it may be interesting to make dietary changes to treat the condition; however, there are no robust studies supporting the use of hypoallergenic diets in the treatment of Chronic Gingivostomatitis in felines. Thus, studies with a higher degree of scientific evidence, assessing cats with gingivostomatitis subjected to elimination diets and provocative exposure, could reinforce the findings of this report that adverse food reactions may be associated with lesions in the oral cavity, and that the use of a hypoallergenic diet could be adjunctive to the treatment of these cases. However, in clinical practice, evaluating the diet of feline patients with gingivostomatitis can be challenging, as these animals often exhibit hyporexia [3,4,9] and may not accept dietary changes, which could leave the patient exposed to potential food antigens. This may explain why many cases of oral lesions in felines are refractory to anti-inflammatory and immunosuppressive treatment, as likely seen in this patient, who did not respond to corticosteroid therapy nor to the first monthly cycle of the most recommended immunosuppressant, cyclosporine [9], even after periodontal treatment.

## 4. Conclusions

The reported case highlights the possibility of an adverse food reaction being a cofactor in the presentation of oral lesions in a feline diagnosed through an elimination diet and provocative exposure, as well as highlighting the benefit of using an hypoallergenic diet based on hydrolyzed protein as an adjunct in treatment.

## Data Availability

The original contributions presented in the study are included in the article, further inquiries can be directed to the corresponding author.
